# Circulating stromal cells in resectable pancreatic cancer correlates to pathological stage and predicts for poor clinical outcomes

**DOI:** 10.1038/s41698-021-00161-8

**Published:** 2021-03-19

**Authors:** Kirby P. Gardner, Mohammed Aldakkak, Cha-Mei Tang, Susan Tsai, Daniel L. Adams

**Affiliations:** 1 Creatv MicroTech, Inc., Monmouth Junction, NJ USA; 2 Rutgers University, Graduate School of Biomedical Sciences, Piscataway, NJ USA; 3 The Medical College of Wisconsin Milwaukee, Milwaukee, WI USA; 4 Creatv MicroTech, Inc., Rockville, MD USA

**Keywords:** Prognostic markers, Pancreatic cancer, Diagnostic markers, Predictive markers

## Abstract

Pancreatic cancer (PC) is notoriously difficult to diagnosis and properly stage resulting in incorrect primary treatment. Diagnostic and prognostic biomarkers are desperately needed to more accurately stage patients and select proper treatments. Recently, a newly discovered circulating stromal cell, i.e. cancer associated macrophage-like cell (CAML), was found to accurately identify solid cancers and predict for worse prognosis. In this pilot study, blood samples were procured from 63 PC patients prior to start of therapeutic intent. CAMLs were found in 95% of samples tested, with ≥12 CAMLs/7.5 mL and ≥50 µm CAMLs both predicting for advanced pathological stage and progression free survival. These data suggest that CAML assessment prior to treatment of PC predicts patients with under-staged disease and with more aggressive PC less likely to respond to standard of care treatment.

## Introduction

Pancreatic cancer (PC) is the third leading cause of cancer mortality in the United States with a predicted 56,770 new cases of PC in 2019 and an estimated 45,750 deaths due to disease^1^. A primary issue causing the high mortality of PC is the inability to diagnose the disease at early stages. ~80% of PC patients are initially diagnosed with unresectable metastatic or locoregional disease while the remaining 20% of earlier staged patients are candidates for surgical resection^2^. In PC, resectability of disease is determined at clinical assessment using CT scans, MRI, PET scans, and Endoscopic ultrasound (EUS)^3^. However, even if a patient is diagnosed with operable PC, only 20% of patients who undergo resection will survive five years^4^. A partial cause of poor 5-year overall survival (OS) is the low accuracy of clinical staging, with ~60% of Stage I PCs being upstaged prior to, or during, primary tumor resection^5^. Upstaging in PC can occur during surgery when evidence of nodal spread or micrometastatic sites are found as a result of pathological assessment, which is more accurate than CT/MRI/PET/EUS scans^5^. Depending on the extent of the upstaging and condition of the tumor, completion of the resection may often not be possible^5^. Sensitive additions to CT Scans, MRI, PET scans, and EUS are needed to more precisely ascertain initial clinical staging that more accurately correlates to pathological staging^6–9^.

Liquid blood biopsies offer the potential to non-invasively identify more aggressive cancer types, but to date, have had very limited success in diagnosing or prognosticating PC^10–12^. While a number of biomarkers can be used in PC, i.e. Carbohydrate antigen 19-9 (CA 19-9) and Carcinoembryonic antigen (CEA), none are highly accurate in diagnosing PC or predicting treatment response^13^. CA 19-9 is a widely used prognostic plasma biomarker (typically considered elevated at ≥37 U/mL) for tracking PC patients response during treatment, however it does not identify cancer spread and cannot be used as a screening biomarker, as it is elevated in non-malignant conditions (i.e. obstructive jaundice)^14^. CEA is plasma biomarker (typically considered elevated at >5 ng/mL) used in monitoring PC over the course of treatment, however, CEA changes can result from non-malignant factors, limiting specificity in monitoring PC^15^. Circulating Tumor Cells (CTC) are tumor cells found in the blood of metastatic cancer patients that identify patients with poorer prognosis, however they are rare in PC and specific to patients with metastatic disease (19%-33% sensitivity of ≥1 CTC/7.5 mL in metastatic PCs)^16,17^. Current blood based biomarkers (i.e. CA 19-9, CEA and CTCs) fall short in assessment of PC disease with generally low sensitivity and low specificity limiting their utility in screening or monitoring disease in the non-metastatic setting^8^.

Cancer associated macrophage-like (CAMLs) cells are a recently identified blood based biomarker found in cancer patients which appear to provide clinical utility in a variety of solid tumor types (i.e., breast, prostate, and lung)^18–20^. CAMLs are giant polynucleated phagocytic myeloid cells which originate from primary tumor sites and have been identified in ~80–100% of early stage cancer patients^20^. Further, CAMLs have been found to not only be prevalent in all stages of cancer, but their number and their increase in size, from phagocytic engorgement, appears be related to staging of disease^18,19^. However to date, no study has evaluated the clinical utility of CAML numbers or CAML size specifically in PC patients. For this study, 63 PC patients who were candidates for surgical resection were asked to volunteer a blood sample for CAML and CTC analysis. Patients were tracked through standard of care neoadjuvant therapy and surgical resection (if surgery was completed), monitored for 24 months to determine the clinical utility of CAML number or CAML size in progression free survival (PFS) or overall survival (OS). In addition, 13 (20%) patients agreed to additional pre-treatment and/or post-treatment blood samples to sequentially monitor changes in CAMLs throughout their therapeutic intervention.

## Results

In total, 85 blood samples were obtained from 63 PC patients. From these 63 PC patients, 13 agreed to a secondary blood draw, seven agreed to a third blood draw, and two agreed to a fourth blood draw. Two baseline blood samples failed due to clotting and insufficient amount of blood (7.5 mL). Of the 63 patients recruited *n* = 62 patients had pancreatic ductal adenocarcinomas and one had pancreatic acinar adenocarcinoma (Table 1). The mean age of the cohort was 66.6 years with a range between 45 and 90 years and interquartile range of 61 and 73.5 years. At clinical diagnosis resectability was assigned based on CT scans. Of these resectability stages, 32% (*n* = 20) were resectable, 43% (*n* = 27) were borderline resectable, 10% (*n* = 6) were locally advanced, and 15% (*n* = 10) were metastatic (Table 1). After neoadjuvant therapy and/or surgical resection 59% (*n* = 37) of patients either progressed or were upstaged. For pathological assessment, 29% (*n* = 18) were stage pI, 19% (*n* = 12) were stage pII, 11% (*n* = 7) were stage pIII, 30% (*n* = 19) were stage pIV, and 11% (*n* = 7) did not have pathological staging due to no surgical resection or dropped off study (Table 1). After 24 months 57%, (*n* = 37/63) of patients experienced disease progression while 43% (*n* = 26/63) did not progress.

**Table 1:** Clinical demographic information for patients.

	(*n* = 63)
Age (Median)	66.6
Age IQR, Range	61–73.5, 45–90
Gender	
Male	37(58%)
Female	26 (41%)
Histology	
Pancreatic adenocarcinoma	62 (98%)
Acinar adenocarcinoma	1 (2%)
Pathological stage	
I	18 (29%)
II	12 (19%)
III	7 (11%)
IV	19 (30%)
Unknown	7 (11%)
Resectability	
Resectable	20 (32%)
Borderline resectable	27 (43%)
Locally advanced	6 (9%)
Metastatic	10 (15%)
CAMLs present	58 (95%)
Median CAML size	57.5 μm (0–189)
CTCs present	14 (22%)
Pre-Surgical CA19-9	
Above threshold (≥37 U/mL)	27 (42%)
Below threshold (<37 U/mL)	31 (49%)
Unknown	5 (7%)
Pre-surgical CEA	
Above threshold (≥5 ng/mL)	10(16%)
Below threshold (<5 ng/mL)	44 (69%)
Unknown	9 (14%)
Neoadjuvant chemo	54 (85%)
Adjuvant chemotherapy	40 (63%)
Radiation therapy	41 (65%)
Surgical resection	37 (58%)
Resection margin	
Positive	3 (4.5%)
Negative	38 (57%)

**Fig. 1:**
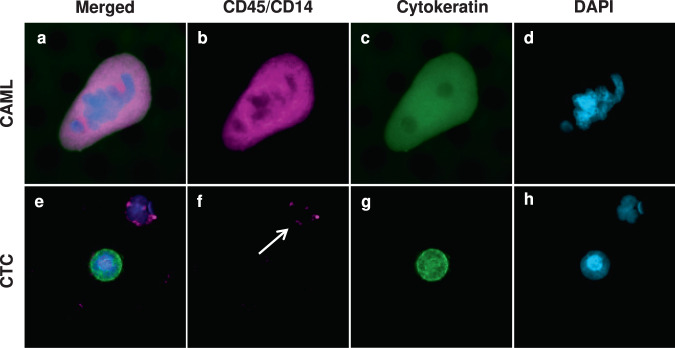
Images of CAML, CTC, and white blood cell. **a**–**d** CAMLs are enlarged cells with a polyploid nucleus (blue), diffuse Cytokeratin (green) and often CD45/CD14 positive (purple). **e**–**h** CTCs are cells with nuclei(blue), filamented Cytokeratin (green), and are CD45/CD14 (purple) negative. White arrow highlights a normal white blood cell (DAPI and CD45/CD14 positive). Boxes = 75 µm.

**Fig. 2:**
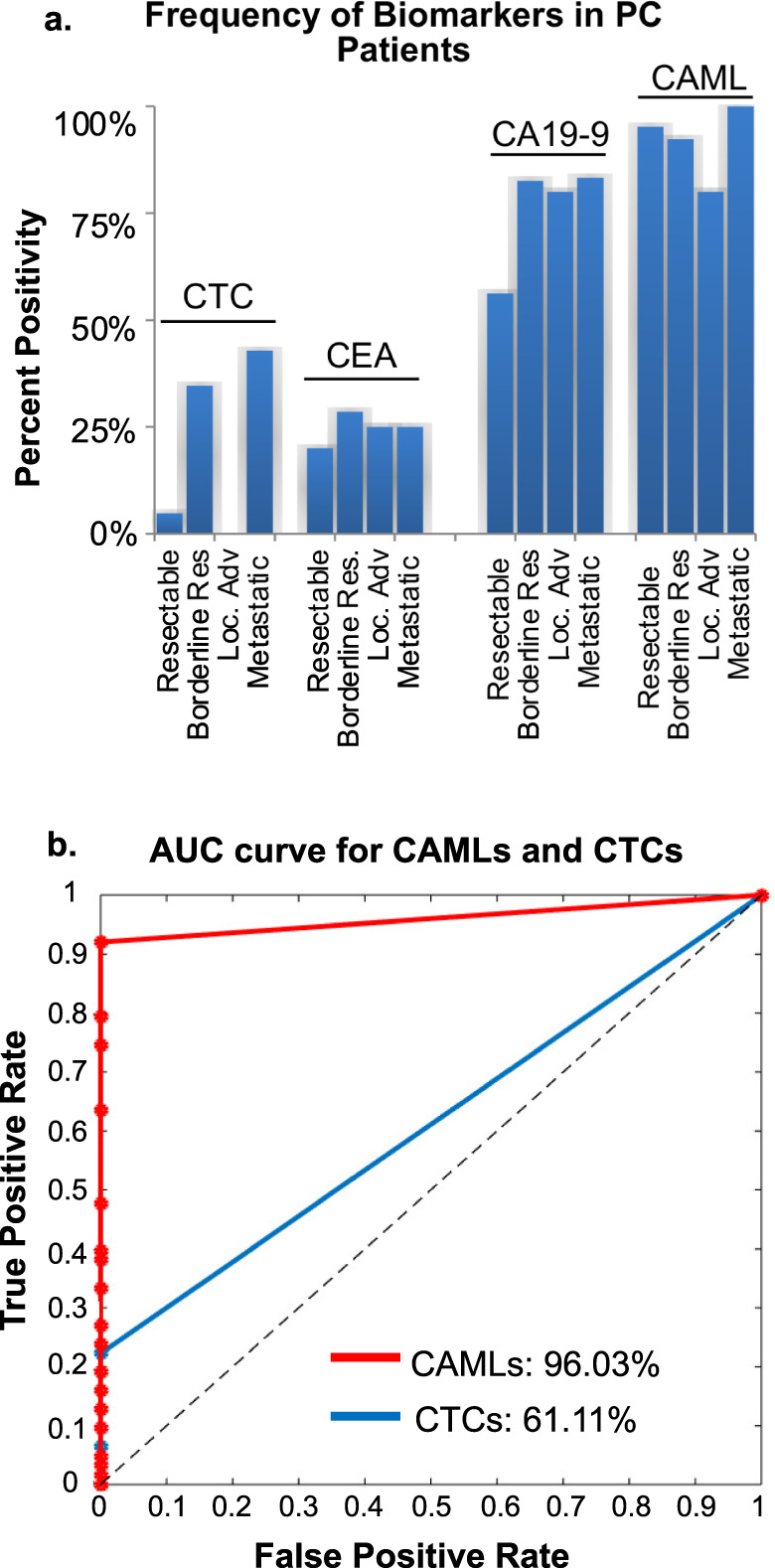
Sensitivity and Specificity of CTCs and CAMLs in Pancreatic cancer patients referred for resection. **a** Frequency of CTCs (>1 cell/sample), CEA (>5 ng/mL), CA19-9 (>37 µg/mL), and CAMLs (>1 cell/sample) as they relate to resectability. **b** ROC curve showing the specificity/sensitivity of CAMLs (red) and CTCs (blue) in comparison with 40 healthy controls.

It has previously been suggested that CAMLs are ubiquitous in all stages of PC^20^. In this study, CAMLs and CTCs were imaged, counted, and analyzed (Fig. 1). CAML positivity was found in 95% (*n* = 58/61) PC patients at baseline and absent in 5% (*n* = 3/61) patients, with (*n* = 2) patients with unknown CAML status due to failures with the samples. CTCs were found in 23% (*n* = 14/61) of PC patients. CAMLs were found in 95% of resectable, 92% of borderline resectable, 80% of locally advanced, and 100% of metastatic PC patients (Fig. 2). In comparison, the healthy control population (*n* = 40) had no CAMLs present in their samples. CA19-9 measurements were available for 58 of the PC patients and CEA measurements were available in 54 of the PC patients (Fig. 2). It was found that measurements of CA19-9 ≥37 U/mL were seen in 42% (*n* = 27/58) of patients while CEA ≥5 ng/mL was seen in 16% (*n* = 10/54) of patients. This suggests the presence of CAMLs appear to be more sensitive than standard cancer biomarkers and future studies must be done to confirm the usefulness of CAMLs as a diagnostic biomarker in comparison to CTCs, CA19-9, and CEA. By comparing CAML sensitivity, it was determined that the ROC curve for healthy controls versus PC patients was found to be AUC = 96% whereas CTCs (AUC = 61%) was far lower (Fig. 2b).

**Fig. 3:**
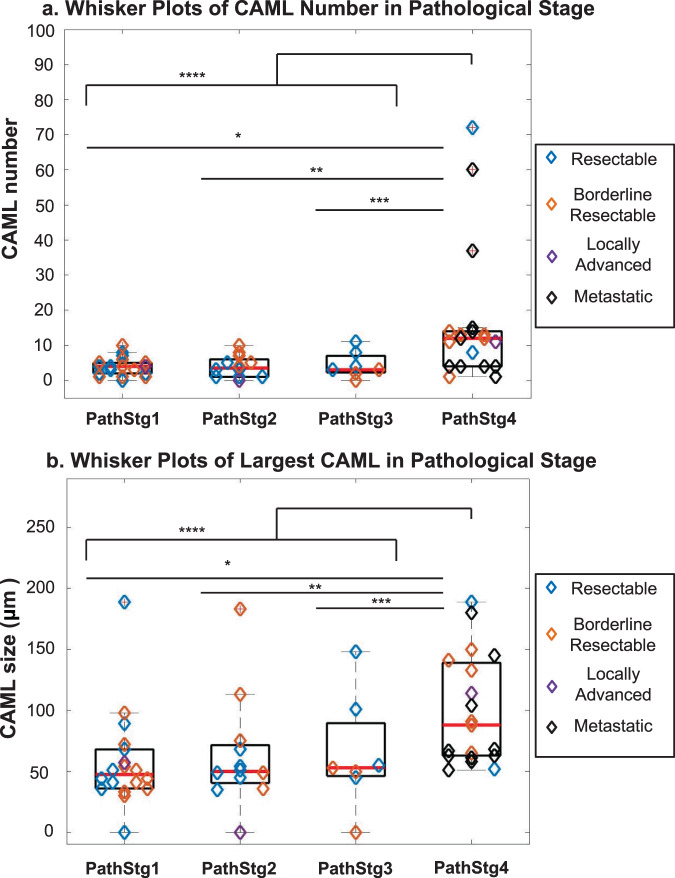
CAML number and Largest CAML size based of Resectability and Pathological stage. **a** Number of CAMLs found in patient baseline blood samples based on Pathological Stage and their original Resectability. Whisker Plots of CAML number based on Pathological Stage (median=red line). Wilcoxon ranked sum test of CAML number Stage 1 vs Stage 4 *p = 0.001, Stage 2 vs Stage 4 **p = 0.003, Stage 3 vs Stage 4 ***p = 0.015, and nonmetastatic vs metastatic disease ****p < 0.001. **b** Largest CAML found in patient baseline blood samples based on Pathological Stage and their original Resectability. Whisker Plots of CAML size based on Pathological Stage (median=red line). Wilcoxon ranked sum of CAML size Stage 1 vs Stage 4 *p < 0.001, Stage 2 vs Stage 4 **p = 0.010, Stage 3 vs Stage 4 ***p = 0.043 and nonmetastatic vs metastatic disease ****p < 0.001.

In previous studies, large CAML size (≥50 µm) and high CAML number have been correlated with later stage disease^19–21^. As PC has a major problem with upstaging, baseline CAMLs were evaluated to see if there was a correlation to the more accurate pathological staging versus initial resectability. CAML number and CAML size (measured as ≥50 µm diameter) taken at clinical assessment were compared against eventual pathological staging after surgery or reimaging after neoadjuvant therapy (Fig. 3). Initial resectability of the disease was unable to fully predict for pIV stage, with only 63% (12/19) of pIV patients being predicted for by clinical scans at diagnosis (Fig. 3a, b). Of the 19 patients who had stage pIV PC, 53% (*n* = 10/19) had ≥12 CAMLs at the baseline time point, while all non-metastatic patients had <12 CAMLs at the baseline time point. Additionally, patients with metastatic disease appeared with larger sizes of CAMLs (median CAML size= 88 μm), while non-metastatic patients had smaller CAMLs (median CAML sizes of 51 μm) (Fig. 3a, b). Using a Wilcoxon ranked sum test, it was determined that number of CAMLs (*p* < 0.001), largest CAML size (*p* < 0.001), average CAML size (*p* = 0.019), presence of CTCs (*p* = 0.011) and elevated CA19-9 (*p* = 0.030) all could differentiate patients with metastatic versus non-metastatic PC (Fig. 3a, b, Supplementary Figures 1, and 2). However, CEA (*p* = 0.261) could not distinguish the same group (Supplementary Figure 2).

**Fig. 4:**
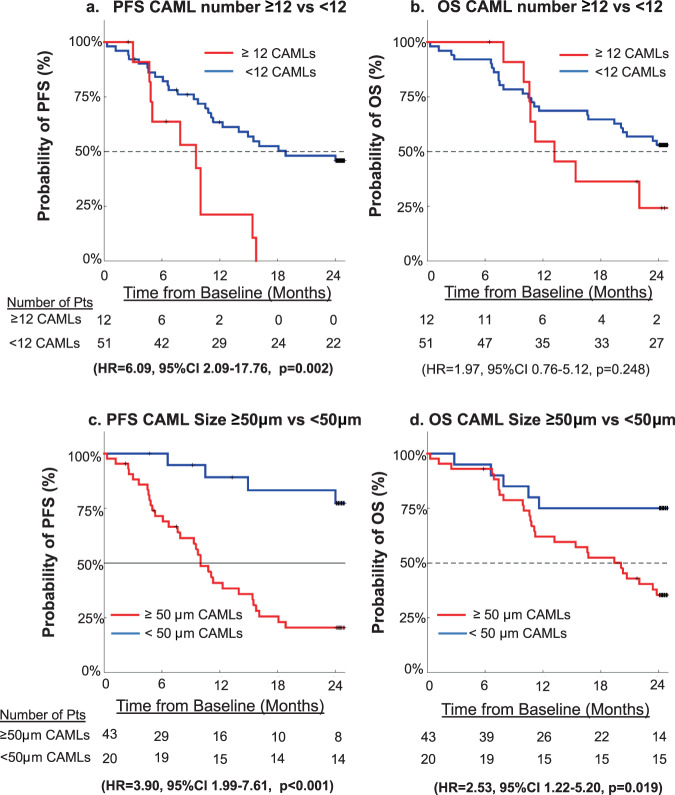
Kaplan–Meier graphs of PFS and OS for CAML number or size. Using ≥ 12 CAMLs as a threshold, Kaplan Meier graphs for **a** PFS and **b** OS. Using ≥ 50 μm as a threshold for largest CAML size, Kaplan–Meier graphs for **c** PFS and **d** OS.

After initial findings that CAMLs appeared correlated with metastatic disease, patients were then monitored for 2 years to compare CAMLs to overall patient’s outcome. Patients with ≥12 CAMLs had a mPFS (median Progression Free Survival) of 8.4 months compared to patients with <12 CAMLs, mPFS of 18.2 months. Patients with ≥12 CAMLs had a mOS (median Overall Survival) of 12.2 months compared to patients with <12 CAMLs not reaching a mOS (Fig. 4). Overall, patients with ≥12 CAMLs had significantly worse outcome than patients <12 CAMLs for PFS (HR = 6.09, 95%CI 2.09–17.76, *p* = 0.002) (Fig. 4a). In addition, the cut-off threshold of ≥11 CAMLs had a slightly higher HR compared to ≥12 CAMLs (e.g., HR_11_ = 6.65 vs HR_12_ = 6.09 for PFS and HR_11_ = 2.12 vs HR_12_ = 1.97 for OS). Though the sensitivity for ≥11 CAMLs was 63.16% vs 52.63% for ≥12 CAMLs, suggesting the ≥12 CAMLs is a more conservative cut-off, but with lower PFS and OS HRs. Further, CAML number was not a significant indicator of OS, with patients ≥12 CAMLs failing to be significantly different than patients with <12 CAMLs (HR = 1.98, 95%CI 0.76–5.12, *p* = 0.248) (Fig. 4a), though there was a trend towards worse outcomes. By comparing size, it was found that patients with CAMLs ≥50μm had mPFS of 9.9 months compared to patients with <50μm CAMLs not reaching mPFS. Additionally, patients with CAMLs ≥50μm had mOS of 19.4 months compared to patients with <50μm CAMLs not reaching mOS. This translated to patients with CAMLs ≥50μm having significantly worse PFS (HR = 3.90, 95%CI 1.99–7.61, *p* < 0.001) and significantly worse OS (HR = 2.53, 95%CI 1.22–5.20, *p* = 0.019) (Fig. 4b). CAML thresholds for CAML number and Largest CAML size, ≥12 CAMLs and ≥50μm respectively, were optimized by comparing all available cut offs by univariate analysis of both PFS and OS (Supplementary Table 1, Supplementary Table 2, Supplementary Table 3, and Supplementary Table 4).

The presence or absence of CTCs, CA19-9 ≥37 U/mL or CEA ≥5 ng/mL all failed to stratify PFS of PC patients (Supplementary Fig. 3 and Supplementary Fig. 4). Similarly, CTC presence, CA19-9 ≥37 U/mL and CEA ≥5 ng/mL also were not significant factors for predicting OS (Supplementary Fig. 3 and Supplementary Fig. 4). This was irregardless of CTC number threshold used as all tested cutoffs failed to predict PFS or (Supplementary Table 5 and Supplementary Table 6). In a multivariate analysis against all known significant variables, it was found that patients that eventually underwent surgical resection (*p* < 0.001) or Largest CAML size at diagnosis (*p* = 0.038) were both significant independent clinical variables for PFS. In a multivariate analysis of OS, it was found that patients that eventually underwent Surgical Resection (*p* < 0.001) or Neoadjuvant therapy (*p* = 0.033) were both significant independent clinical variables (Supplementary Table 7). Interestingly, the fact that CAML number was not a significant indicator in the multivariate analysis was a result from a relationship between CAML number and CAML size. This is not surprising as the presence of larger CAMLs is related to the presence of any CAMLs, though is worthy of future study to evaluate the biological underpinnings of why CAML size is such a stronger indicator for PFS, than their presence.

**Fig. 5:**
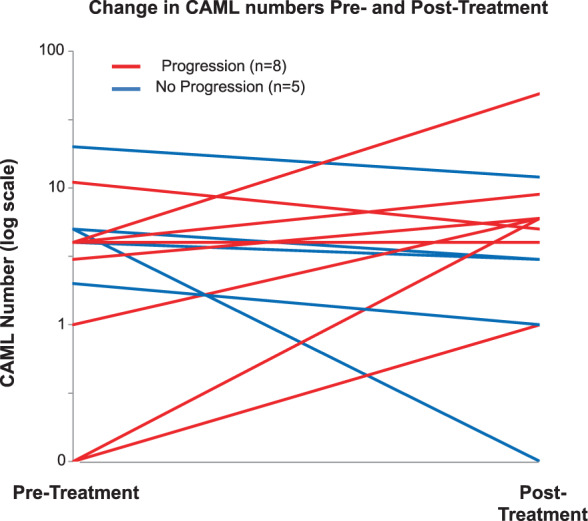
CAML enumeration for patients that progressed within 2 years versus patients that did not progress. Patients who volunteered for multiple blood draws during the course of treatment (*n* = 13). Pre-Treatment and post-treatment time points were taken. Patients that experienced progression during a 2 year period (red lines), with six patients having an increase in CAML number, one patient having equal numbers, and one having a decrease in CAML number from 11 to 5 CAMLs. Patients that did not experience progression during a 2 year period (blue lines), with all five non-progressing patients had a decrease in CAML number.

In addition to baseline blood sampling, thirteen (21%) patients volunteered to give additional sequential blood draws with blood draws performed both pre- and post-treatment (Fig. 5). While preliminary pilot findings, *n* = 6/8 patients who experienced progression had an increase in CAML number in their post-surgical blood samples, one patient had the same number of CAMLs, and one patient saw a decrease from 11 to 5 CAMLs (Fig. 5). In contrast, all patients (*n* = 5) who had experienced no progression had a decrease in CAML number. Similar patterns were not observed while tracking CA19-9, CEA, largest CAML size change and CTCs change as it related to patients progression (Supplementary Fig. 5 and Supplementary Fig. 6).

## Discussion

In this study analyzing the clinical utility of CAMLs in pancreatic cancer, it was determined that CAMLs are commonly found in both pre-treated and treatment naïve patient samples in all stages of disease. At initial diagnosis, presence of CAMLs appeared to act as a sensitive blood biomarker of PC disease with patients having ≥12 CAMLs being highly indicative of advanced metastatic disease, regardless of initial clinical diagnosis. Further, the presence of either high CAML number (≥12) or large (≥50 µm) appeared to significantly correlate for worse PFS, with (≥50 µm) CAML size also correlating for worse OS. Overall, in a multivariate analysis of all available clinical parameters, surgical resection and largest CAML were found to be the most significant independent predictor of progression free survival. For overall survival, it was found that surgical resection and resectability were the most significant independent predictors of overall survival. These results support the hypothesis that largest CAMLs may act as a biomarker for identifying patients with PC, but also as a clinically predictive tool to identify patients likely to progress with highly aggressive PC.

CAMLs were not found in any healthy controls indicating that CAMLs may exist exclusively in active neoplasms, like PC disease. The finding of CAMLs in 92% of PC, and none in healthy controls is a promising result and suggest a possible screening method to identify PC. While intriguing, expanded studies must be undertaken to confirm our findings and further, to evaluate CAMLs in relation to premalignant and non-malignant medical conditions that may mimic PC. Premalignant lesions such as pancreatic intraepithelial neoplasia (PanIN) and Intraductal papillary mucinous neoplasms (IPMN) are theorized to progress into pancreatic adenocarcinoma in a stepwise manor analogous to polyps and colon cancer^2^, with macrophages often being recruited to these premalignant lesions^22,23^. As CAMLs have shown CD14 + , and are theorized to be myeloid origin based on their phagocytic macrophage-like nature^18–20^, it can be surmised that they may also recruit to these premalignant lesions in the course of progression to pancreatic exocrine tumors. Screening for PanIN or IPMN is especially important in people with familial pancreatic cancer, Peutz-Jeghers syndrome, familial atypical multiple mole melanoma syndrome, and genetic mutations like PALB2 or BRCA2^9^. If CAMLs show similar prevalence in premalignant lesions, surgeons can identify and resect these lesions and prevent the development of PC entirely, though well-developed screening studies are needed.

These results illustrate the potential for using CAMLs for differentiating between metastatic (i.e. pathological stage IV) and non-metastatic disease (pathological stage I-III) (Fig. 3), and for identifying patients more likely to progress using standard clinical treatments. As an example, a single patient with high numbers of CAMLs (72 CAMLs at baseline) was diagnosed with resectable PC (Fig. 3). The patient’s CA19-9 measurements had a decrease from 18.4 U/mL to 6.0 U/mL and the patient completed neoadjuvant chemotherapy and underwent surgical resection. However, within 10 months from the baseline blood draw, the patient progressed with metastatic disease and expired. In this particular case, CAML enumeration could have supplemented the other biomarkers to present a more complete picture of the aggressiveness and spread of the disease. A PC patient can only undergo CT scans roughly every few months, and these scans can be both a financial burden and general health risk to the patient^8,24^. It has been previously established that CT and EUS are inefficient at imaging smaller metastatic sites, resulting in surgical upstaging of patients^5^, which was also found in this study. Recently, there has been a push for less invasive and costly medical tests to evaluate the responsiveness of a patient to various treatments^8^. These results suggest that CAMLs may be a non-invasive blood test that adds a simple addition to more accurately assess PC patients. These tests might be used as a tool to minimize unnecessary surgical procedures for more appropriate medical interventions. By predicting response and outcomes using CAMLs, testing the effectiveness of treatments might be possible more frequently and with much less financial or health burden to the patient. Oncologists may be able to predict disease progression even faster than standard scans, accurately tracking PC and the efficacy of treatments to allow doctors to alter courses of treatment and potentially save lives.

There is a potential to determine a patient’s likelihood to progress and expire from PC by analysis of largest CAML found at baseline. Similarly, by enumerating CAMLs in peripheral blood at baseline, a physician could potentially be able to determine a patient’s chances to progress during the course of treatment. This in conjunction with information from CA 19-9 measurements and clinical scans could provide oncologists with a more comprehensive understanding of the of progression and potential lethality of disease. Similarly based on our findings on monitoring CAML numbers after therapeutic interventions, a physician could determine the probability of progression/relapse post-surgical resection. In Fig. 5, we found that patients who experienced either spikes in CAML numbers from baseline to post therapeutic interventions experienced progression, while those who did not progress experienced a decline in CAML number. However, as these findings are based on a small pilot population and while interesting initial findings, further testing is needed to validate if CAML enumeration is a potential monitoring tool for PC.

If these findings hold true, this could revolutionize the treatment of PC, giving insight to the physician for patients that are unlikely to benefit from surgical resection and requires aggressive chemotherapeutic intervention for maximizing patient outcomes. CAMLs might greatly reduce incidence of upstaging events, predict a patient’s likelihood to progress during the course of treatment, and determine a patient’s responsiveness to surgical resection. Although CAMLs relationship between the spread of pancreatic cancer is yet to be fully illustrated, these giant myeloid cells might prove invaluable in better understanding, diagnosing, and prognosticating this fatal disease.

## Methods

### Cohort recruitment

A prospective single blind pilot study was initiated to evaluate the presence and prognostic value of CAMLs found in the peripheral blood of PC patients. Peripheral blood samples were obtained from 63 PC patients through collaboration with the Medical College of Wisconsin, in accordance with the local institutional review board (IRB) approval and with the patient’s informed written consent. PC patients were recruited from 2012 to 2014, having been referred for pancreatic resection based on clinical evaluation. Patients were classified as having resectable (*n* = 20), borderline resectable (*n* = 27), locally advanced (*n* = 6) PC; or patients scheduled for primary resection after previous treatment for metastatic disease (*n* = 10). Of the cohort, 62 patients had pancreatic ductal adenocarcinoma tumor histology and one patient had pancreatic acinar adenocarcinoma. At clinical diagnosis, scans of the patients were used to determine resectability (resectable, borderline resectable, locally advanced, and metastatic) of disease. Pathological stage was determined after surgical resection. For this study, pathological stage IV were defined as metastatic at diagnosis, pathologically metastatic based on surgical resection, or expired from advanced disease prior to resection. Peripheral blood was obtained from all 63 patients at the time of diagnosis prior to any treatment. Additionally, blood samples were procured pre- and post-surgical treatment from *n* = 13 of the 63 patients. Patients were monitored for 2 years after initial blood sample draws with the clinical variables age, gender, resectability, pathological staging if available, neoadjuvant and adjuvant therapy (chemotherapy and radiation therapy), tumor markers (CA199 and CEA) and evaluated (Table 1). Anonymized blood samples were collected into CellSave preservation vacutainers (Menarini-Silicon Biosystems) and shipped within 96 hours for processing. Patient’s clinical information was not shared or unblinded until the completion of the study. In addition, blood samples from 40 age matched healthy control volunteers, including 16 females (40%) and 24 males (60%) with an average age of 64.4 years old, were collected in CellSave tubes according to local IRB (Western Institutional Review Board) approval and with informed consent.

### Tumor associated circulating cell capture

Samples were run with a CellSieve Microfiltration Assay using a low-pressure vacuum system^18–20^. CellSieve Microfiltration isolates CTCs and CAMLs via size exclusion. The identification of the CTCs is done by morphological feature as well as the expression of EpCAM, cytokeratin 8, 18, and 19, and DAPI; with absence of CD45. CAMLs were identified by their enlarged polynucleated (14-64 micrometer diameter) nucleus and by their enlarged cellular body (21 to 300 micrometers in length) which is often positive for a diffuse cytokeratin expression and/or CD45 expression. For the assay briefly, 7.5 mL of blood was prefixed for 15 min placed into the 30 mL syringe and drawn through the filter at a preset pressure. After the blood was filtered, microfilters were washed with 6 mL of PBS followed by postfixation for 15 min and placed in permeabilization buffer for 15 min^19^. The filter and cells were stained using an antibody mixture and blocking buffer mixture containing FITC conjugated cytokeratin 8, 18, 19 antibody, phycoerythrin (PE)-conjugated EpCAM antibody, and a Cy5-conjugated CD45 antibody from a 1x concentration of CellSieve Enumeration Stain Solution (Creatv Microtech) After staining, filters were washed with 10 mL of PBS + 0.1% Tween-20 (PBST) and mounted onto a glass microscope slide using Flouromount-G with DAPI (Southern Biotech).

### Analysis of filters

After samples underwent filtration and staining protocol listed above, samples underwent CTC and CAML enumeration. An Olympus BX54WI Fluorescent microscope with a Carl Zeiss AxioCam was used for the imaging of CTCs and CAMLs (Fig. 1). The Zen2011 Blue computer program was used to process the images. Cell size was measured using the precalibrated size tools in the Zen2011 Blue software.

### Statistical analysis

Cox proportional hazard regression were used to determine the univariate and multivariate hazard ratios with a statistical analysis’s threshold of *p* ≤ 0.05, using MATLAB R2013A using all available clinical parameters from all patients. Wilcoxon ranked sum tests were used to determine p values in comparing CAML numbers in stages (Fig. 2). PFS and OS Kaplan–Meier estimation was done using the time to progression defined as the interval between when blood sample was obtained, to the date of progression by standard RECIST criteria using PET/CT scan or death, within 24-month end point.

### Reporting summary

Further information on research design is available in the Nature Research Reporting Summary linked to this article.

## Supplementary information

Supplementary Figures PDF

REPORTING SUMMARY

## Data Availability

The data supporting the findings of this study, are publicly available in the figshare repository: 10.6084/m9.figshare.13660916^25^. The original cell images supporting Fig. 1, will be made available on reasonable request from Mr. Kirby Gardner, email address: kgardner@creatvmicrotech.com. The data generated and analyzed during this study are described in the following metadata record: 10.6084/m9.figshare.13809992^26^.

## Code availability

Statistical analysis was done using MATLAB R2020b (The Mathworks Inc, Natick, Massachusetts, USA). Codes for statistical analysis were KMplot (Cardillo, 2021)^27^, Logrank (Cardillo, 2021)^28^, and ROC curve (Cardillo, 2021)^29^.
