# Supplementary material for: Circulating stromal cells in resectable pancreatic cancer correlates to pathological stage and predicts for poor clinical outcomes
Source: NPJ Precis Oncol. 2021 Mar 19;5:25. doi: 10.1038/s41698-021-00161-8 (PMC7979885; doi:10.1038/s41698-021-00161-8)
Supplement: Supplementary file 1 — Supplementary Figures PDF [file 41698_2021_161_MOESM1_ESM.pdf]

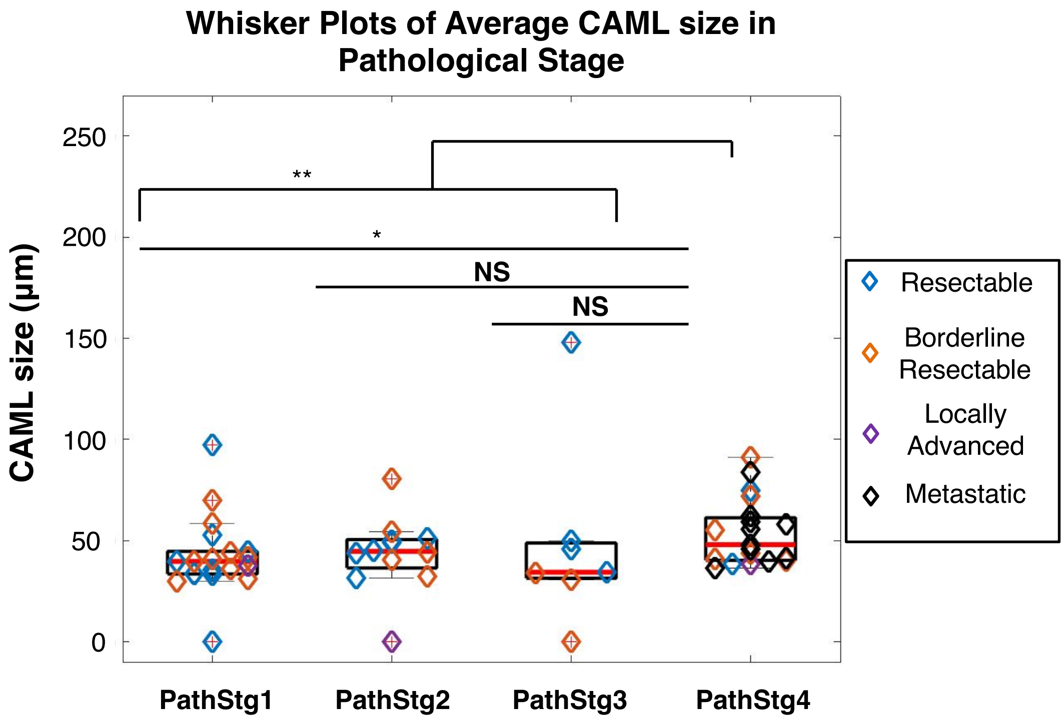

Supplementary Figure 1: Average CAML size based on Resectability and Pathological stage. Average CAML size found in patient baseline blood samples based on Pathological Stage and their original Resectability. Whisker Plots of CAML number based on Pathological Stage (median=red line). Wilcoxon ranked sum test of CAML number Stage 1 vs Stage 4 *p=0.018 and non-metastatic vs metastatic disease**p=0.019**.** Both Stage 2 vs Stage 4 (p=0.247) and Stage 3 vs Stage 4 (p=0.099) were found to be non-significantly (NS) different.

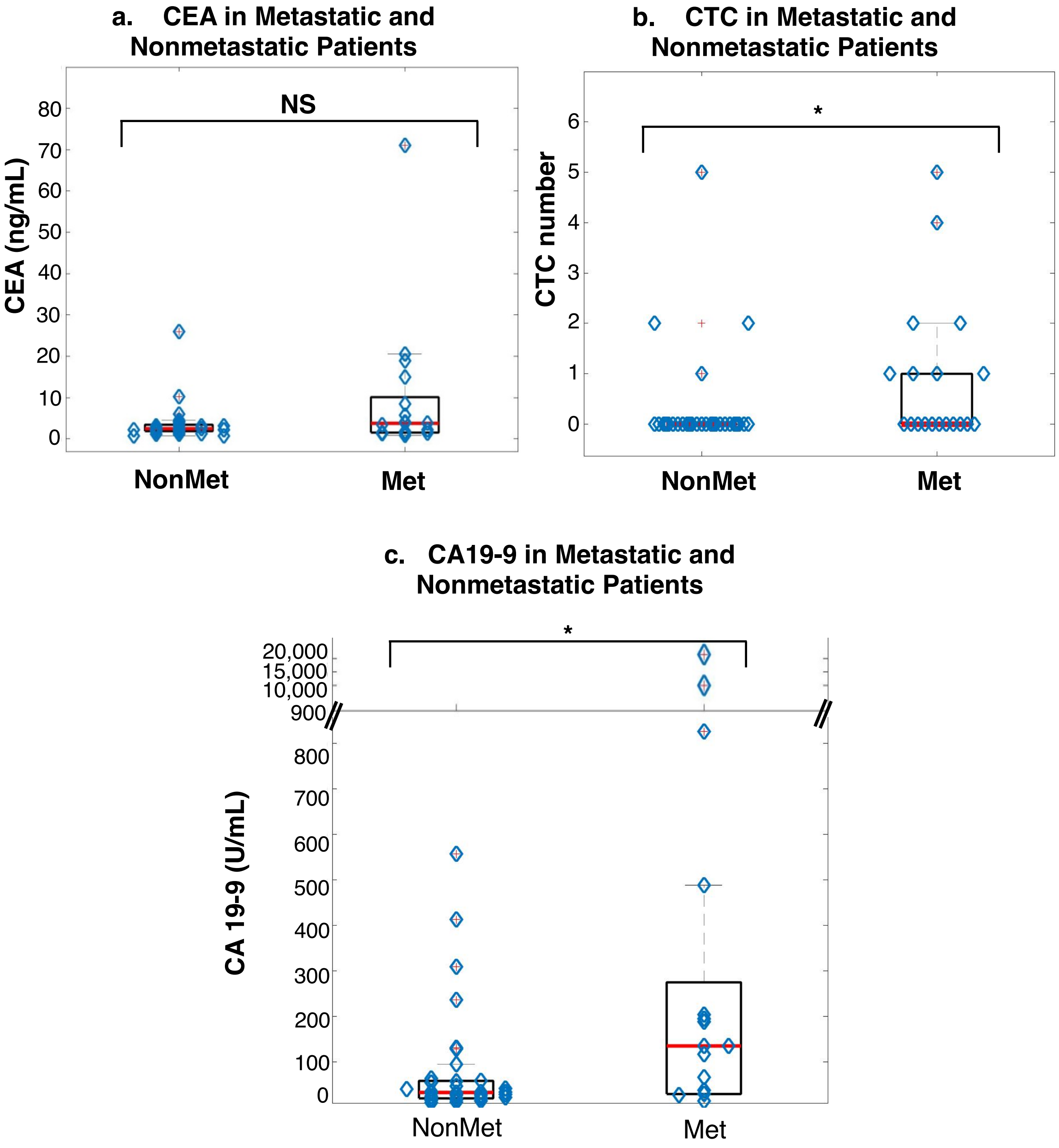

Supplementary Figure 2: Comparison of various biomarkers in metastatic vs nonmetastatic PC. **a.** Whisker Plots of CEA number based on Metastasis (median=red line). Wilcoxon Ranked Sum Test (WT) of CEA number non-metastatic (NonMet) vs metastatic (Met) disease was nonsignificant (NS) p=0.261**. b.** Whisker Plots of CTC number based on Metastasis (median=red line). WT of CTC number NonMet vs Met disease was significant p=0.011 **c.** Whisker Plots of CA19-9 number based on Metastasis (median=red line). WT of CEA number NonMet vs Met disease was significant p=0.030

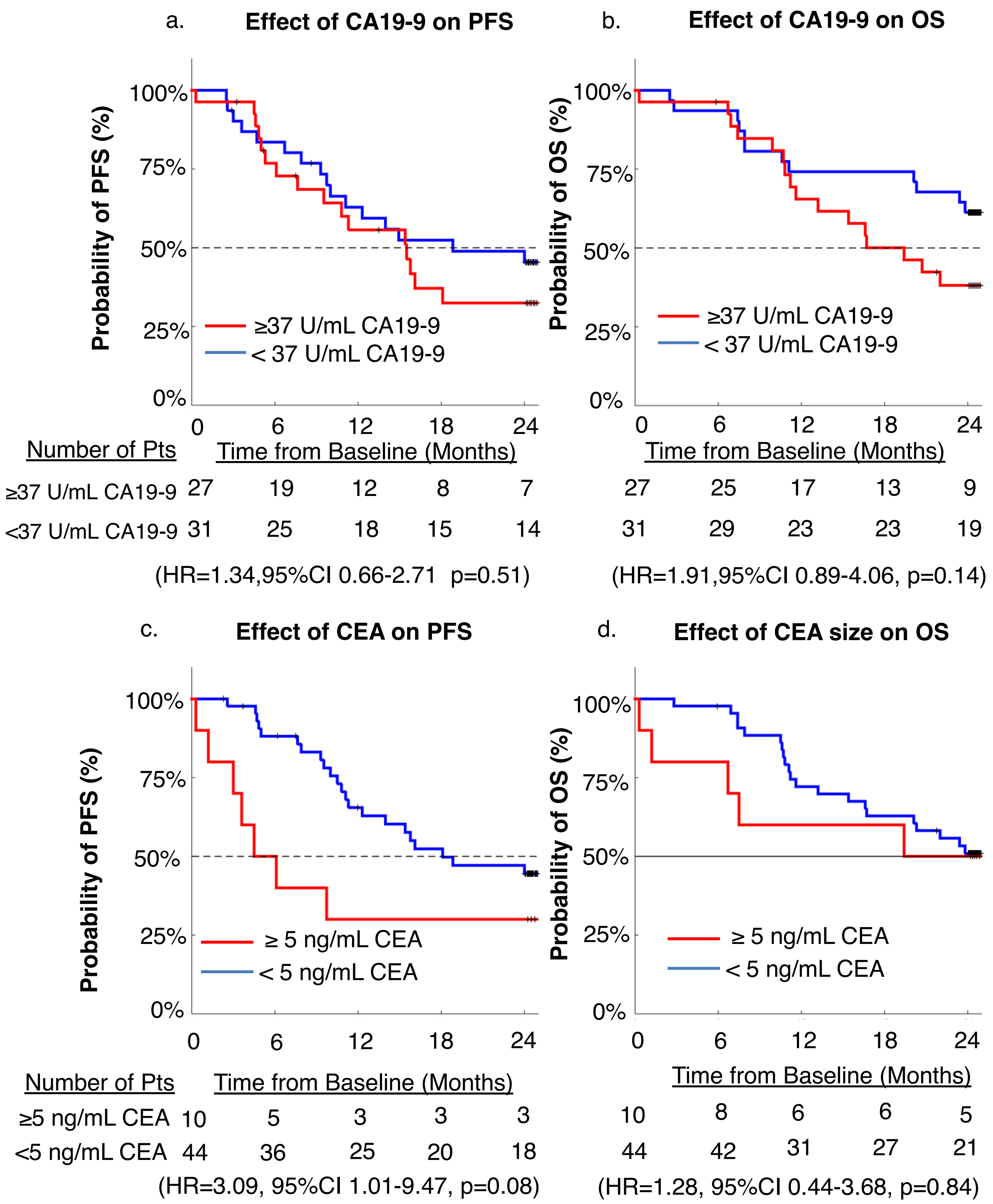

Supplementary Figure 3: Kaplan-Meirer graphs of PFS and OS for CA19-9 ≥ 37 U/mL and CEA ≥ 5 ng/mL

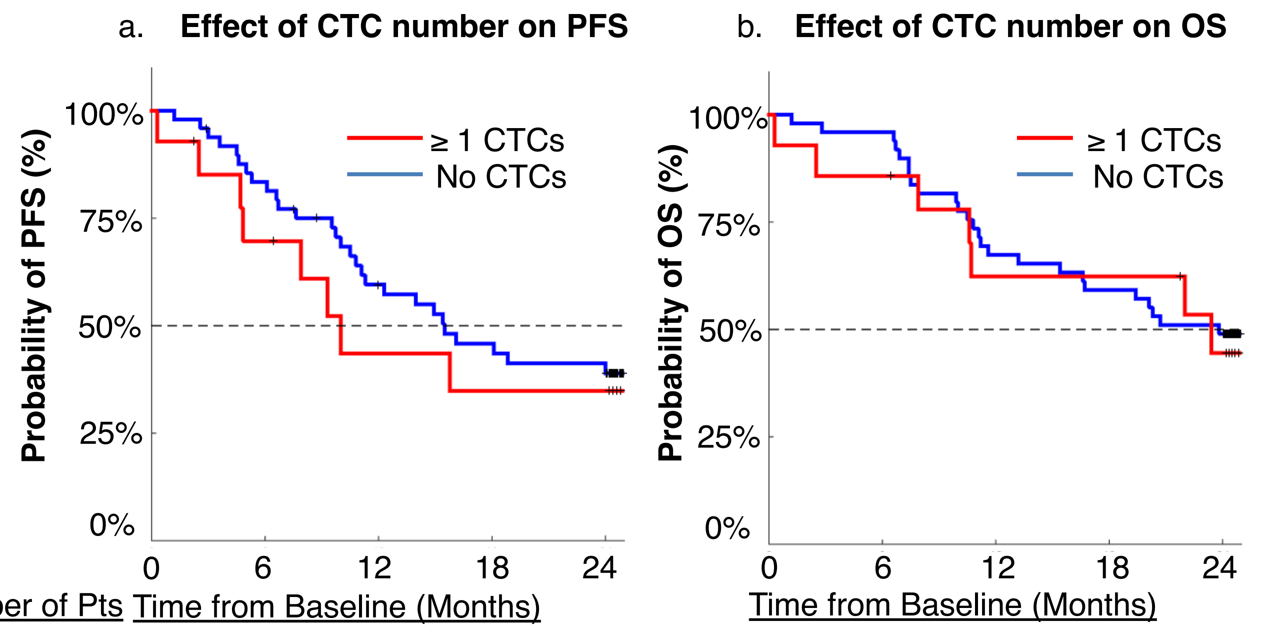

Number of Pts										
≥1 CTCs	14	8	5	4	4	14	11	8	8	5
No CTC	49	40	26	20	18	49	48	34	30	25

(HR=1.37, 95%CI 0.58-3.25, p=0.61) (HR=1.10, 95%CI 0.46-2.60, p=0.99)

Supplementary Figure 4: Kaplan-Meier graphs of PFS and OS for CTCs

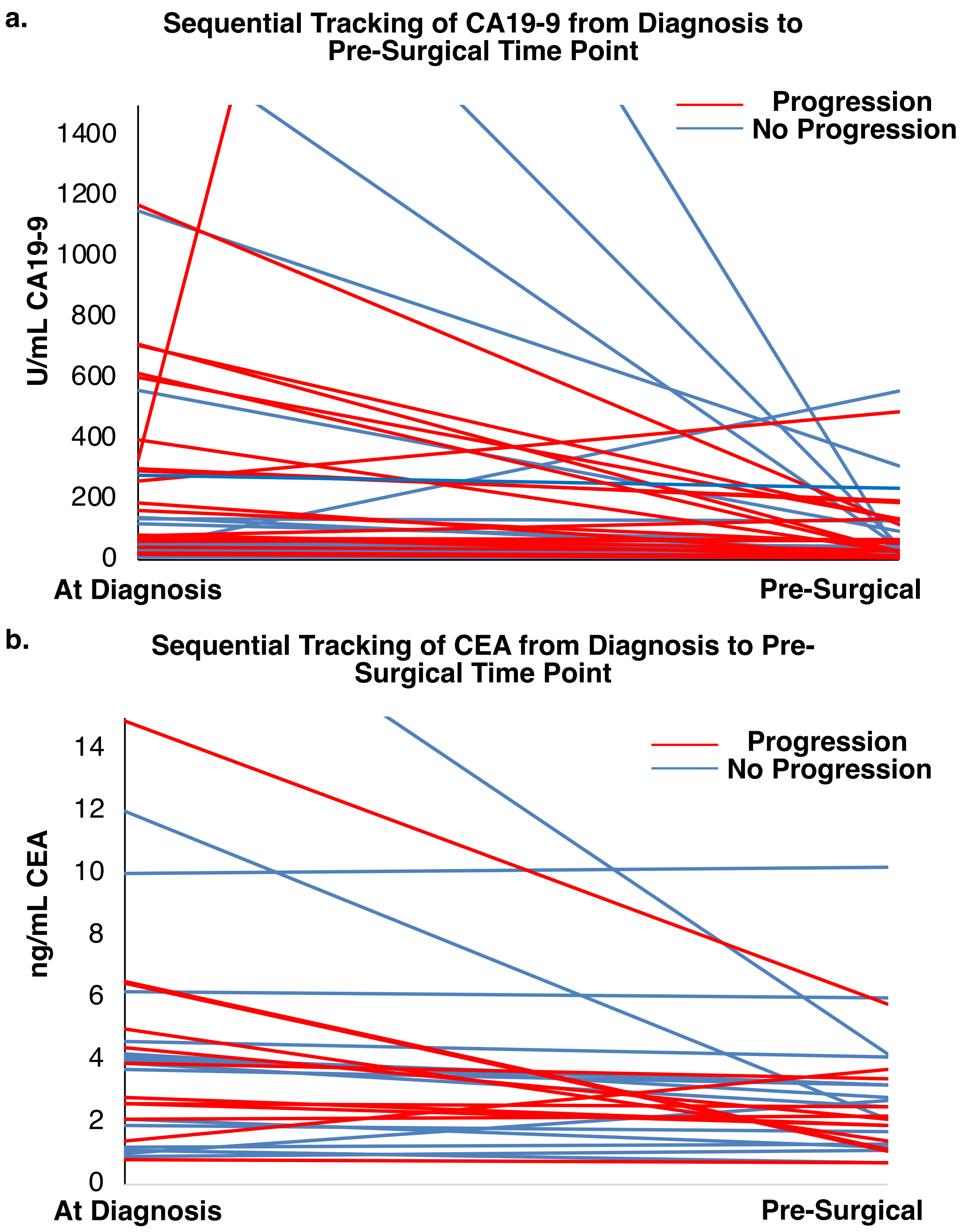

Supplemental Figure 5: Sequentially Tracking CA19-9 and CEA from Diagnosis to Pre-Surgical Time Point. a. 41 patients had CA19-9 measurements at both diagnosis and pre-Surgical time points. 20 Patients did not experience progression, with 18 decreasing in CA19-9 and 2 increasing in CA19-9). 21 Patients experienced progression, with 17 decreasing CA19-9 and 4 increasing CA 19-9). **B.** 28 pts with CEA measurements at both diagnosis and pre-Surgical time points. 17 patients experienced no progression, with 13 decreasing in CEA and 4 increasing in CEA). 11 patients experienced progression with 9 decreasing CEA and 2 increasing CEA.

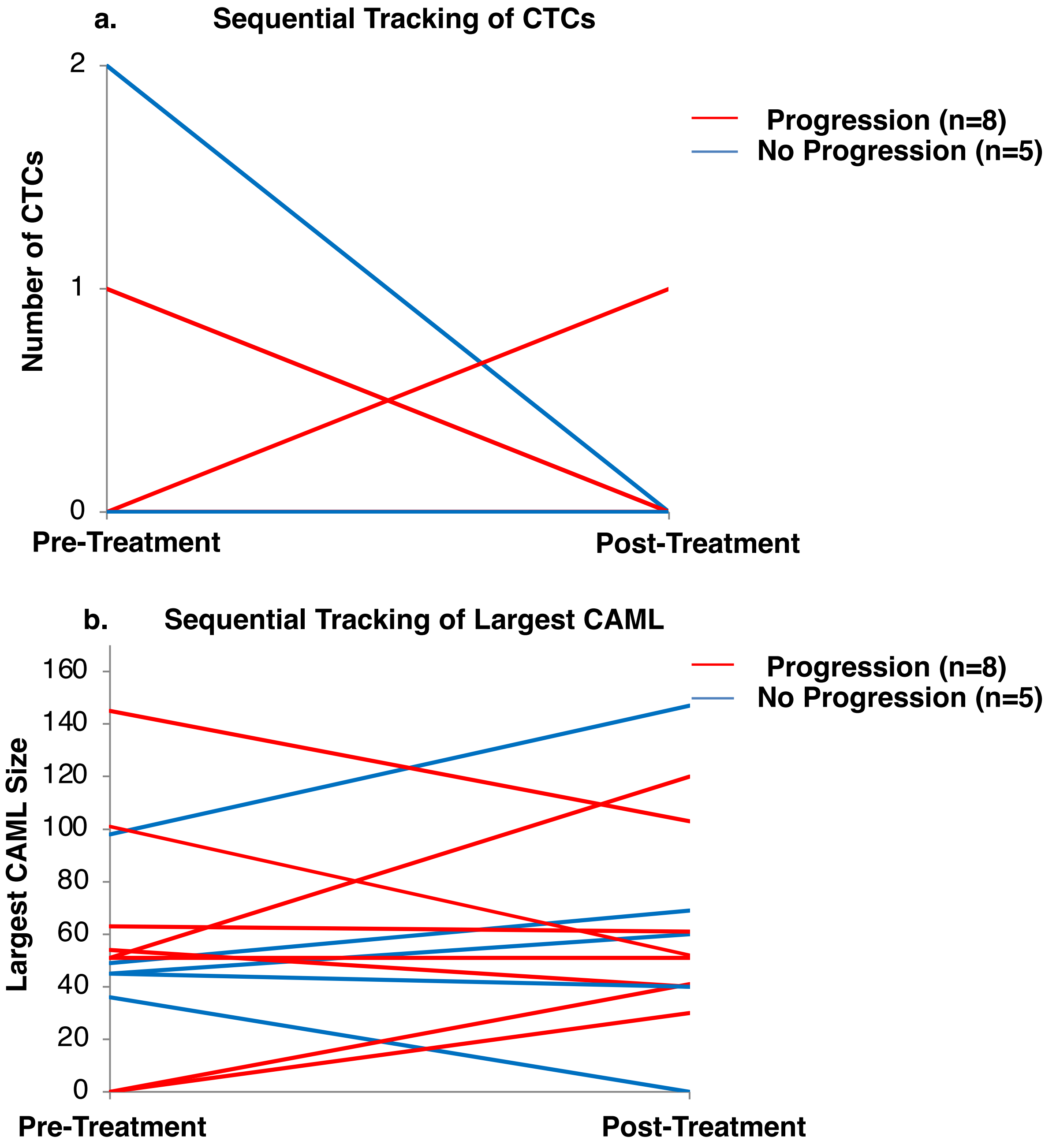

Supplemental Figure 6: CTC enumeration and Largest CAML Measurements for patients that progressed within 2 years versus patients that did not progress. Patients who volunteered for multiple blood draws during the course of treatment (n=13). Pre-treatment and post-surgical time points were taken. Each line is comparing Pre-treatment and post-treatment time points only per patient. **a.** Number of CTCs Pre- and Post-treatment. Seven patients had zero CTCs at both time points, three patients had one CTC at Pre-treatment and zero CTCs at Post-treatment, and two patients had two CTCs at Pre-treatment and zero CTCs at Post-treatment. **b.** Largest CAML Size Pre- and Post-surgery.

n (number of CAMLs)	Pts with < n	Pts with ≥ n	HR	CI	p value
1	4	58	0.78	0.16-3.85	0.91
2	12	50	1.35	0.61-2.98	0.58
3	15	47	1.52	0.73-3.16	0.34
4	22	40	1.29	0.66-2.53	0.55
5	32	30	1.20	0.62-2.32	0.70
6	37	25	1.80	0.90-3.61	0.13
7	38	24	1.83	0.90-3.71	0.13
8	41	21	2.41	1.13-5.16	0.03
9	45	17	2.96	1.28-6.83	0.01
10	45	17	2.96	1.28-6.83	0.01
11	47	15	6.65	2.54-17.40	<0.01
12	50	12	6.09	2.09-17.76	<0.01
13	52	10	5.79	1.90-17.61	<0.01
14	54	8	3.75	`1.17-11.97	0.05
15	56	6	4.78	1.29-17.66	0.04
16	59	3	4.14	0.69-24.70	0.26

Supplementary Table 1: Table of potential thresholds for CAML number compared to PFS. Hazard ratios (HR), Confidence Intervals (CI), and p values included for each different number (n). Significant values highlighted in bold.

n (number of CAMLs)	Pts with < n	Pts with ≥ n	HR	CI	p value
1	4	58	0.02	0.003-0.22	<0.01
2	12	50	0.99	0.40-2.43	0.83
3	15	47	1.15	0.51-2.59	0.88
4	22	40	0.87	0.41-1.82	0.86
5	32	30	0.81	0.40-1.65	0.70
6	37	25	1.30	0.63-2.69	0.59
7	38	24	1.23	0.59-2.57	0.69
8	41	21	1.49	0.69-3.22	0.40
9	45	17	1.49	0.65-3.39	0.44
10	45	17	1.49	0.65-3.39	0.44
11	47	15	2.12	0.87-5.13	0.14
12	50	12	1.97	0.76-5.12	0.24
13	52	10	1.95	0.71-5.37	0.29
14	54	8	2.30	0.74-7.10	0.24
15	56	6	3.23	0.86-12.12	0.16
16	59	3	12.69	1.48-108.58	0.07

Supplementary Table 2: Table of various thresholds for CAML number compared to OS. Hazard ratios (HR), Confidence Intervals (CI), and p values included for each different number (n). Significant values highlighted in bold.

n (Largest CAML in microns)	Pts with < n	Pts with ≥ n	HR	CI	p value
30	4	58	0.78	0.16-3.85	0.91
40	10	52	2.05	0.90-4.67	0.13
50	19	43	3.90	1.99-7.61	<0.01
60	32	30	2.43	1.23-4.80	0.01
70	42	20	2.20	1.02-4.73	0.06
80	44	18	2.38	1.07-5.29	0.5
90	46	16	2.72	1.16-6.37	0.03
100	48	14	3.24	1.30-8.08	0.02
110	51	11	2.57	0.94-7.01	0.11
120	54	9	2.05	0.69-6.08	0.30
130	54	9	2.05	0.69-6.08	0.30
140	55	8	1.60	0.53-4.83	0.57
150	58	5	1.04	0.31-3.46	0.80

Supplementary Table 3: Table of various thresholds for Largest CAML size compared to PFS. Hazard ratios (HR), Confidence Intervals (CI), and p values included for each different number (n). Significant values highlighted in red.

n (Largest CAML in microns)	Pts with < n	Pts with ≥ n	HR	CI	p value
30	4	58	0.027	0.003-0.22	<0.01
40	10	52	1.30	0.50-3.36	0.76
50	19	43	2.53	1.22-5.20	0.019
60	32	30	1.91	0.93-3.90	0.11
70	42	20	1.17	0.55-2.51	0.81
80	44	18	1.15	0.53-2.51	0.86
90	46	16	1.17	0.52-2.62	0.85
100	48	14	1.24	0.53-2.91	0.77
110	51	11	1.16	0.45-2.95	0.94
120	54	9	1.34	0.46-3.87	0.77
130	54	9	1.34	0.46-3.87	0.77
140	55	8	1.03	0.35-3.02	0.84
150	58	5	0.73	0.21-2.55	0.87

Supplementary Table 4: Table of various potential thresholds for Largest CAML size compared to OS. Hazard ratios (HR), Confidence Intervals (CI), and p values included for each different number (n). Significant values highlighted in red.

n (number of CTCs)	Pts with < n	Pts with ≥ n	HR	CI	p value
1	48	14	1.37	0.58-3.23	0.61
2	54	8	0.85	0.32-2.25	0.93
3	58	4	1.41	0.36-5.45	0.87
4	59	3	3.85	0.66-22.25	0.28
5	60	2	2.21	0.33-14.47	0.72

Supplementary Table 5: Table of various thresholds for CTC compared to PFS. Hazard ratios (HR), Confidence Intervals (CI), and p values included for each different number (n).

n (number of CTCs)	Pts with $< n$	Pts with $\geq n$	HR	CI	p value
1	48	14	1.14	0.47-2.75	0.93
2	54	8	0.85	0.31-2.31	0.96
3	58	4	1.38	0.36-5.30	0.89
4	59	3	2.50	0.51-12.33	0.46
5	60	2	1.74	0.30-10.11	0.86

Supplementary Table 6: Table of various potential thresholds for CTC compared to OS. Hazard ratios (HR), Confidence Intervals (CI), and p values included for each different number (n).

	Univ. HR(PFS)	Univ. p value (PFS)	Multi. p value (PFS)	Univ. HR (OS)	Univ. p value (OS)	Mult. p value (OS)
Clinical Stage						
Clin 1 v Clin 4	0.21	0.44	*	<0.01	**0.02**	*
Clin 1 v Clin2	0.08	**<0.01**	*	0.15	**<0.01**	*
Clin 2 v Clin 4	1.19	0.89	*	0.61	0.94	*
Non Met v Met	0.39	0.65	*	0.03	0.11	*
Clin 1 v Clin 1 & Clin 4	0.08	**<0.01**	0.65	0.18	**<0.01**	0.06
Resectability						
Res v BR	0.41	0.07	*	0.68	0.54	*
Res v LA	<0.01	**<0.01**	*	0.57	0.75	*
Res v Met	0.04	**<0.01**	*	0.06	**<0.01**	*
BR v LA	0.26	0.12	*	0.91	0.84	*
BR v Met	0.22	**0.01**	*	0.11	**<0.01**	*
LA v Met	1.01	0.79	*	0.24	**0.03**	*
R & BR v LA & Met	**0.10**	**<0.01**	0.48	**0.27**	**<0.01**	**0.03**
Surgery	22.7	**<0.01**	**<0.01**	5.85	**<0.01**	**<0.01**
Age	1.04	0.96	*	0.94	0.98	*
Gender	1.18	0.87	*	0.71	0.43	*
Largest CAML	3.89	**<0.01**	**0.03**	2.53	0.17	0.30
CAML number	6.09	**<0.01**	0.16	1.97	0.24	*
CTC number	1.37	0.61	*	1.10	0.99	*
Received Neoadjuvant	1.99	0.39	*	7.324	**0.07**	**0.03**
Received Chemotherapy	0.69	0.38	*	1.58	0.30	*
Received Radiation Therapy	1.89	0.12	*	2.05	0.09	*
CA19-9(≥ 37U/mL)	1.34	0.51	*	1.91	0.14	*
CEA (≥ 5 ng/mL)	3.09	0.08	*	1.28	0.84	*

Supplementary Table 7: Univariate and Multivariate analysis for clinical variables available. Univariate analysis was run for each individual clinical variable. Clinical stage univariates were run amongst stages, with Clinical Stage 1 vs Clinical Stage 2 & Clinical Stage 4 found to be the most significant. No patients were Clinical Stage 3 and therefore not included. Resectability was found to be significant between the stages, most significant being a combination of Resectable (R) and Borderline Resectable (BR) compared to Locally Advanced (LA) and Metastatic (Met). All factors found to be significant ($p<0.075$) were included in multivariate analysis. For PFS, Surgery and Largest CAMLs were significant. For OS, Surgery, neoadjuvant therapy, and resectability were significant. Worth noting Surgery was determined after treatment, whereas other factors were determined prior to treatment.
