# Supplementary material for: Circulating stromal cells in resectable pancreatic cancer correlates to pathological stage and predicts for poor clinical outcomes
Source: NPJ Precis Oncol. 2021 Mar 19;5:25. doi: 10.1038/s41698-021-00161-8 (PMC7979885; doi:10.1038/s41698-021-00161-8)
Supplement: Supplementary file 2 — REPORTING SUMMARY [file 41698_2021_161_MOESM2_ESM.pdf]

nature research

Corresponding author(s): Dan Adams

Last updated by author(s): Jan 28, 2021

Reporting Summary

Nature Research wishes to improve the reproducibility of the work that we publish. This form provides structure for consistency and transparency in reporting. For further information on Nature Research policies, see our Editorial Policies and the Editorial Policy Checklist.

Statistics

For all statistical analyses, confirm that the following items are present in the figure legend, table legend, main text, or Methods section.

n/a	Confirmed	
☐	☒	The exact sample size (n) for each experimental group/condition, given as a discrete number and unit of measurement
☐	☒	A statement on whether measurements were taken from distinct samples or whether the same sample was measured repeatedly
☐	☒	The statistical test(s) used AND whether they are one- or two-sided<br>*Only common tests should be described solely by name; describe more complex techniques in the Methods section.*
☐	☒	A description of all covariates tested
☐	☒	A description of any assumptions or corrections, such as tests of normality and adjustment for multiple comparisons
☐	☒	A full description of the statistical parameters including central tendency (e.g. means) or other basic estimates (e.g. regression coefficient) AND variation (e.g. standard deviation) or associated estimates of uncertainty (e.g. confidence intervals)
☐	☒	For null hypothesis testing, the test statistic (e.g. F, t, r) with confidence intervals, effect sizes, degrees of freedom and P value noted<br>*Give P values as exact values whenever suitable.*
☒	☐	For Bayesian analysis, information on the choice of priors and Markov chain Monte Carlo settings
☒	☐	For hierarchical and complex designs, identification of the appropriate level for tests and full reporting of outcomes
☒	☐	Estimates of effect sizes (e.g. Cohen's d, Pearson's r), indicating how they were calculated

Our web collection on statistics for biologists contains articles on many of the points above.

Software and code

Policy information about availability of computer code

Data collection	*Provide a description of all commercial, open source and custom code used to collect the data in this study, specifying the version used OR state that no software was used.*
Data analysis	Statistical analysis was done using MATLAB R2020b (The Mathworks Inc, Natick, Massachusetts, USA). Codes for statistical analysis were: KMplot (https://github.com/dnafinder/kmplot), Logrank (https://github.com/dnafinder/logrank), and AUC/ROC curve (https://github.com/dnafinder/roc)

For manuscripts utilizing custom algorithms or software that are central to the research but not yet described in published literature, software must be made available to editors and reviewers. We strongly encourage code deposition in a community repository (e.g. GitHub). See the Nature Research guidelines for submitting code & software for further information.

Data

Policy information about availability of data

All manuscripts must include a data availability statement. This statement should provide the following information, where applicable:

- Accession codes, unique identifiers, or web links for publicly available datasets
- A list of figures that have associated raw data
- A description of any restrictions on data availability

The data that support the findings of this study are openly available at:

DOI: 10.6084/m9.figshare.13660916

All other data are available from the corresponding author upon request.

Field-specific reporting

Please select the one below that is the best fit for your research. If you are not sure, read the appropriate sections before making your selection.

☒ Life sciences ☐ Behavioural & social sciences ☐ Ecological, evolutionary & environmental sciences

For a reference copy of the document with all sections, see nature.com/documents/nr-reporting-summary-flat.pdf

Life sciences study design

All studies must disclose on these points even when the disclosure is negative.

Sample size	63 Pancreatic Cancer patients from Medical College of Wisconsin. 85 samples with 13 patients agreeing to multiple blood draws
Data exclusions	2 baseline samples failed due to clotting and insufficient amount of blood
Replication	This is a pilot study
Randomization	patients samples were anonymized
Blinding	Patient's clinical information was not shared or unblinded until the completion of the study.

Reporting for specific materials, systems and methods

We require information from authors about some types of materials, experimental systems and methods used in many studies. Here, indicate whether each material, system or method listed is relevant to your study. If you are not sure if a list item applies to your research, read the appropriate section before selecting a response.

Materials & experimental systems

n/a	Involved in the study	
☐	☒	Antibodies
☒	☐	Eukaryotic cell lines
☒	☐	Palaeontology and archaeology
☒	☐	Animals and other organisms
☐	☒	Human research participants
☐	☒	Clinical data
☒	☐	Dual use research of concern

Methods

n/a	Involved in the study	
☒	☐	ChIP-seq
☒	☐	Flow cytometry
☒	☐	MRI-based neuroimaging

Antibodies

Antibodies used	Pre-diluted antibody mixture containing FITC conjugated cytokeratin 8, 18, 19 antibody, phycoerythrin (PE)-conjugated EpCAM antibody, and a Cy5-conjugated CD45 antibody from manufacturer (used at a 1x concentration per manufacturer's instructions).
Validation	Antibody validation and applications have been previously established in several publications (see citations 18-20 of manuscript). Link to antibody database and validation available at http://www.creatvmicrotech.com/enumeration.html.

Human research participants

Policy information about studies involving human research participants

Population characteristics	Peripheral blood samples were obtained from 63 PC patients through collaboration with the Medical College of Wisconsin, in accordance with the local institutional review board (IRB) approval and with the patient's informed consent. PC patients were recruited from 2012 to 2014, having been referred for pancreatic resection based on clinical evaluation. Patients were classified as having resectable (n=20), borderline resectable (n=27), locally advanced (n=6) PC; or patients scheduled for primary resection after previous treatment for metastatic disease (n=10). Of the cohort, 62 patients had pancreatic ductal adenocarcinoma tumor histology and one patient had pancreatic acinar adenocarcinoma.Additionally, blood samples were procured pre- and post-surgical treatment from n=13 of the 63 patients.The mean age of the cohort was 66.6 years with a range between 45 and 90 years and interquartile range of 61 and 73.5 years. At clinical diagnosis resectability was assigned based on CT scans. Of these resectability stages, 32% (n=20) were resectable, 43% (n=27) were borderline resectable, 10% (n=6) were locally advanced, and 15% (n=10) were metastatic (Table 1). After neoadjuvant therapy and/or surgical resection 59% (n=37) of patients either progressed or were upstaged. For pathological assessment, 29% (n=18) were stage pI, 19% (n=12) were stage pII, 11% (n=7) were stage pIII, 30% (n=19) were stage pIV, and 11% (n=7) did not have pathological staging due to no surgical resection or dropped off study (Table 1). After 24 months 57%, (n=37/63) of patients experienced disease progression while 43% (n=26/63) did not progress. In addition, blood samples from 40 age matched healthy control

	volunteers, including 16 females (40%) and 24 males (60%) with an average age of 64.4 years old, were collected in CellSave tubes according to local IRB (Western Institutional Review Board) approval and with informed consent
Recruitment	Peripheral blood samples were obtained from 63 PC patients through collaboration with the Medical College of Wisconsin, in accordance with the local institutional review board (IRB) approval and with the patient's informed consent. PC patients were recruited from 2012 to 2014, having been referred for pancreatic resection based on clinical evaluation.
Ethics oversight	Peripheral blood samples were obtained from 63 PC patients through collaboration with the Medical College of Wisconsin, in accordance with the local institutional review board (IRB) approval and with the patient's informed consent.

Note that full information on the approval of the study protocol must also be provided in the manuscript.

Clinical data

Policy information about clinical studies

All manuscripts should comply with the ICMJE guidelines for publication of clinical research and a completed CONSORT checklist must be included with all submissions.

Clinical trial registration	*Provide the trial registration number from ClinicalTrials.gov or an equivalent agency.*
Study protocol	*Note where the full trial protocol can be accessed OR if not available, explain why.*
Data collection	Peripheral blood samples were obtained from 63 PC patients through collaboration with the Medical College of Wisconsin, in accordance with the local institutional review board (IRB) approval and with the patient's informed consent. PC patients were recruited from 2012 to 2014, having been referred for pancreatic resection based on clinical evaluation. In addition, blood samples from 40 age matched healthy control volunteers, including 16 females (40%) and 24 males (60%) with an average age of 64.4 years old, were collected in CellSave tubes according to local IRB (Western Institutional Review Board) approval and with informed consent.
Outcomes	PFS and OS Kaplan-Meier estimation was done using the time to progression defined as the interval between when blood sample was obtained, to the date of progression by standard RECIST criteria using PET/CT scan or death, within 24-month end point.
